# Supplementary material for: Modulating the PPARγ pathway upregulates NECTIN4 and enhances chimeric antigen receptor (CAR) T cell therapy in bladder cancer
Source: Nat Commun. 2025 Sep 10;16:8215. doi: 10.1038/s41467-025-62710-0 (PMC12423289; doi:10.1038/s41467-025-62710-0)
Supplement: Supplementary file 1 — Supplementary Information [file 41467_2025_62710_MOESM1_ESM.pdf]

Supplementary Figure 1:

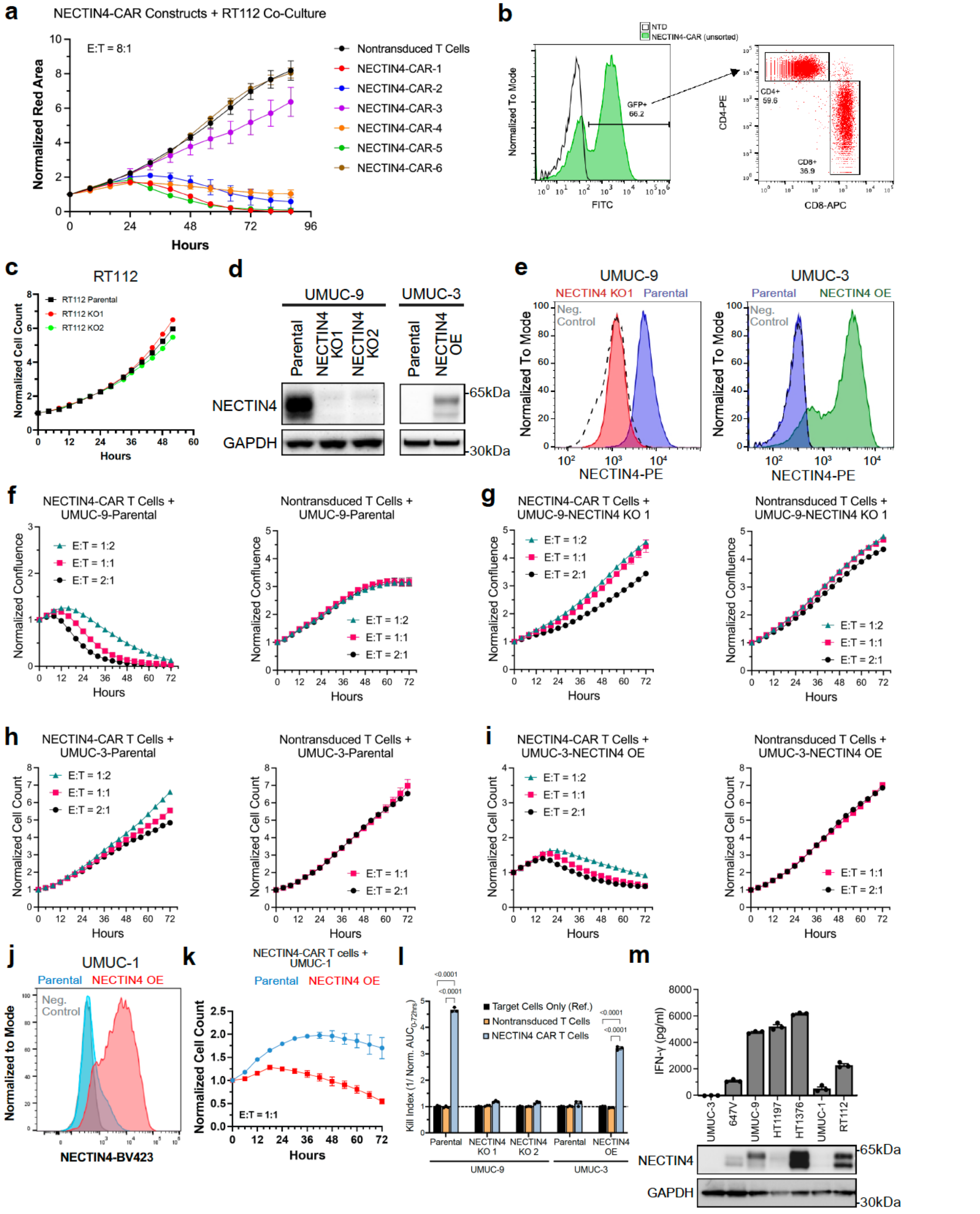

Supplementary Figure 1: Screening second-generation NECTIN4-CAR constructs to identify a lead for CAR T cell therapy demonstrating NECTIN4-specific killing. (**a**) Normalized growth curves of NECTIN4^{MED}-expressing RT112 target cells over time co-cultured with T cells expressing CARs containing different NECTIN4 scFv sequences. Screening experiments were performed at an effector-to-target (E:T) ratio of 8:1. Data are presented as mean +/- SEM for each time point. (**b**) Flow cytometry analysis of T cells expressing NECTIN4-

CAR-1 from (**a**). CAR expression was identified by GFP. T cells were stained for surface CD4 and CD8. Histogram shows NTD T cells (black open) overlaid with NECTIN4-CAR-transduced (green filled) T cells with CD4+ and CD8+ subpopulations are indicated. (**c**) Cell proliferation assay of RT112 parental and 2 *NECTIN4* knockout (KO) clones. A representative experiment performed in triplicate wells of n=3 biologically independent experiments is shown, and similar results were obtained. (**d**) Western blots showing NECTIN4 expression in $NECTIN4^{HIGH}$ UMUC-9 parental cells and NECTIN4 KO cells and in $NECTIN4^{NEG}$ UMUC-3 parental cells and NECTIN4 overexpressing (OE) cells. GAPDH was used as a protein loading control. This blot was repeated n=3 independent times with similar results. (**e**) NECTIN4 surface protein expression in UMUC-9 parental and NECTIN4 KO cells (left) and in the UMUC-3 parental and NECTIN4 OE cells (right) overlaid with unstained controls (dotted open). **(f-g)** Growth curves of (**f**) UMUC-9 parental and (**g**) UMUC-9 NECTIN4 KO target cells co-cultured with NECTIN4-CAR (left) or NTD (right) T cells at indicated E:T ratios. **(h-i)** Growth curves of (**h**) UMUC-3 parental and (**i**) UMUC-3 NECTIN4 OE target cells co-cultured with NECTIN4-CAR (left) or NTD (right) T cells at indicated E:T ratios. **(j)** NECTIN4 surface protein expression in UMUC-1 (blue) and UMUC-1 NECTIN4 OE (red) cells. The negative unstained control is shown in gray. **(k)** Growth curves of UMUC-1 parental and UMUC-1 NECTIN4 OE target cells co-cultured with NECTIN4-CAR T cells at an E:T ratio of 1:1. **(l)** Kill index of NECTIN4-CAR T cells against indicated target cells at an E:T ratio of 1:1. A two-way ANOVA with Sidak's multiple comparison test was used. (**m**) IFN-γ quantification by ELISA from co-cultures of NECTIN4-CAR T cells with a panel of human UC cell lines with variable expression of NECTIN4 as shown on western blot (bottom) at 24h. This blot was repeated n=2 independent times with similar results. Data are presented as mean +/- SEM, n=3 biological replicates. For panels **f-i,** and **k-m**, data are presented as mean +/- SEM, n=3 biological replicates. For panels **f-i,** and **k**, data are presented as mean +/- SEM for each time point, and a representative of n=3 biologically independent experiments performed in technical triplicates is shown. Source data are provided as a Source Data file.

Supplementary Figure 2:

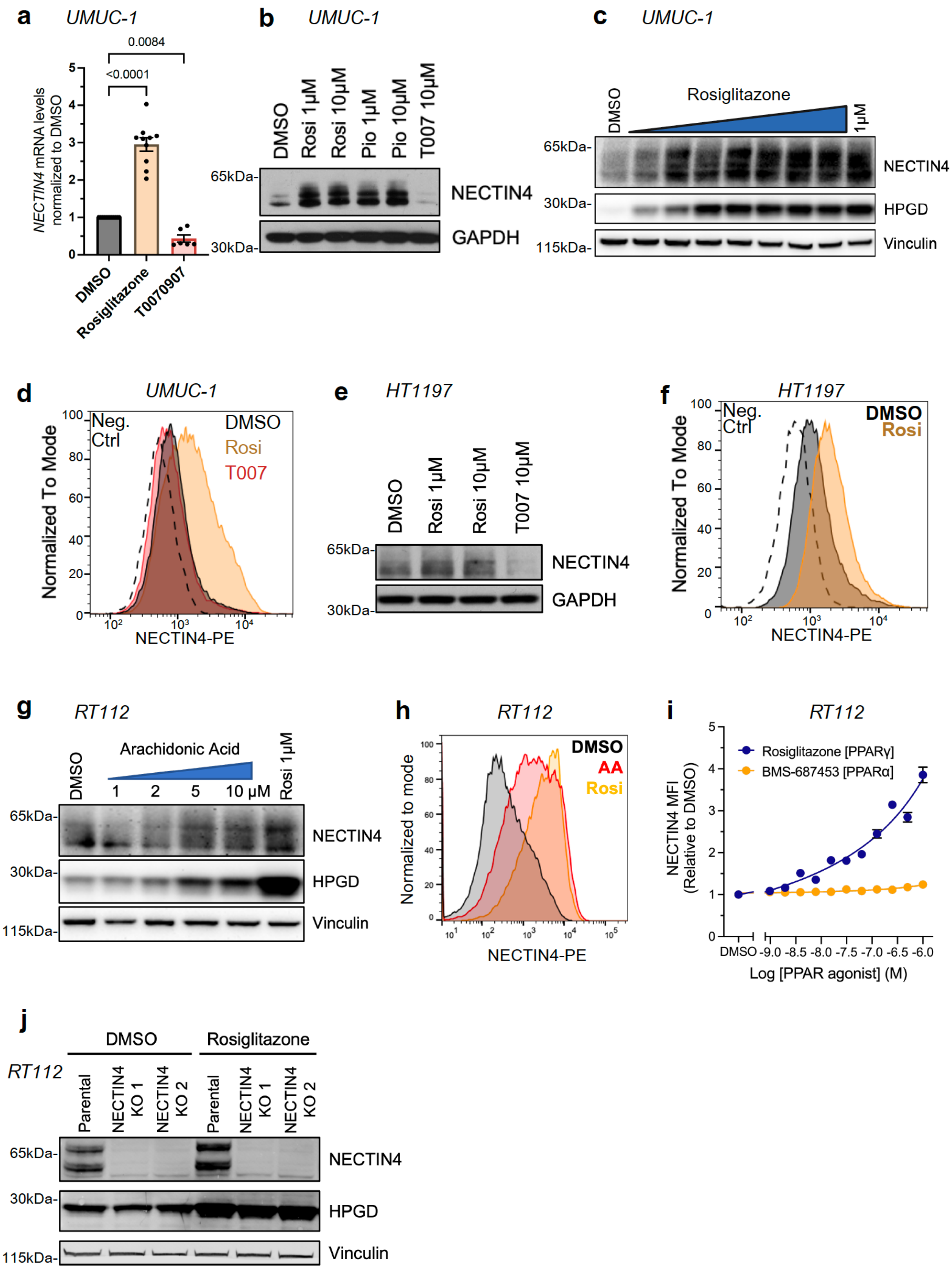

Supplementary Figure 2: PPARγ-specific modulation of NECTIN4 expression across multiple UC cell lines. (**a**) *NECTIN4* mRNA levels in UMUC-1 cells treated with 1 µM rosiglitazone or 1 µM T0070907 for 72h. Data are presented as mean +/- SEM, n=12 biological replicates for DMSO and rosiglitazone, n=6 biological replicates for T0070907, and a two-way ANOVA with Sidak's multiple comparison test was used. (**b**) Western blot for NECTIN4 in UMUC-1 cells treated with T0070907 (T007), rosiglitazone (Rosi), and pioglitazone (Pio) at

indicated concentrations for 72h. (**c**) Western blot for NECTIN4 and HPGD in UMUC-1 cells treated with rosiglitazone for 72h across a dose series (starts at 1 μM on the right-most lane with serial 2-fold dilutions). (**d**) Surface staining for NECTIN4 protein in UMUC-1 cells treated with 1 μM rosiglitazone or 1 μM T0070907 for 72h. (**e**) Western blot for NECTIN4 in HT1197 cells treated with rosiglitazone and T0070907 at indicated concentrations for 72h. **(f)** Surface staining for NECTIN4 protein in HT1197 cells treated with 10 μM rosiglitazone for 72 hours. (**g**) Western blot for NECTIN4 and HPGD in RT112 cells treated with arachidonic acid (AA) and rosiglitazone at indicated concentrations for 72h. (**h**) Surface staining for NECTIN4 protein in RT112 cells treated with 10 μM AA (red) or 1 μM rosiglitazone (yellow) for 72h. (**i**) Dose response curves of surface NECTIN4 expression in RT112 cells treated with rosiglitazone (navy, data from Fig. 2g) compared to with PPARα-specific agonist BMS-687453 (yellow) for 72h. Data are presented as mean +/- SEM. (**j**) Western blot for NECTIN4 and HPGD in RT112 parental and NECTIN4 KO cells following treatment with 1 μM rosiglitazone for 72h. All blots were repeated n=3 independent times with similar results. Source data are provided as a Source Data file.

Supplementary Figure 3:

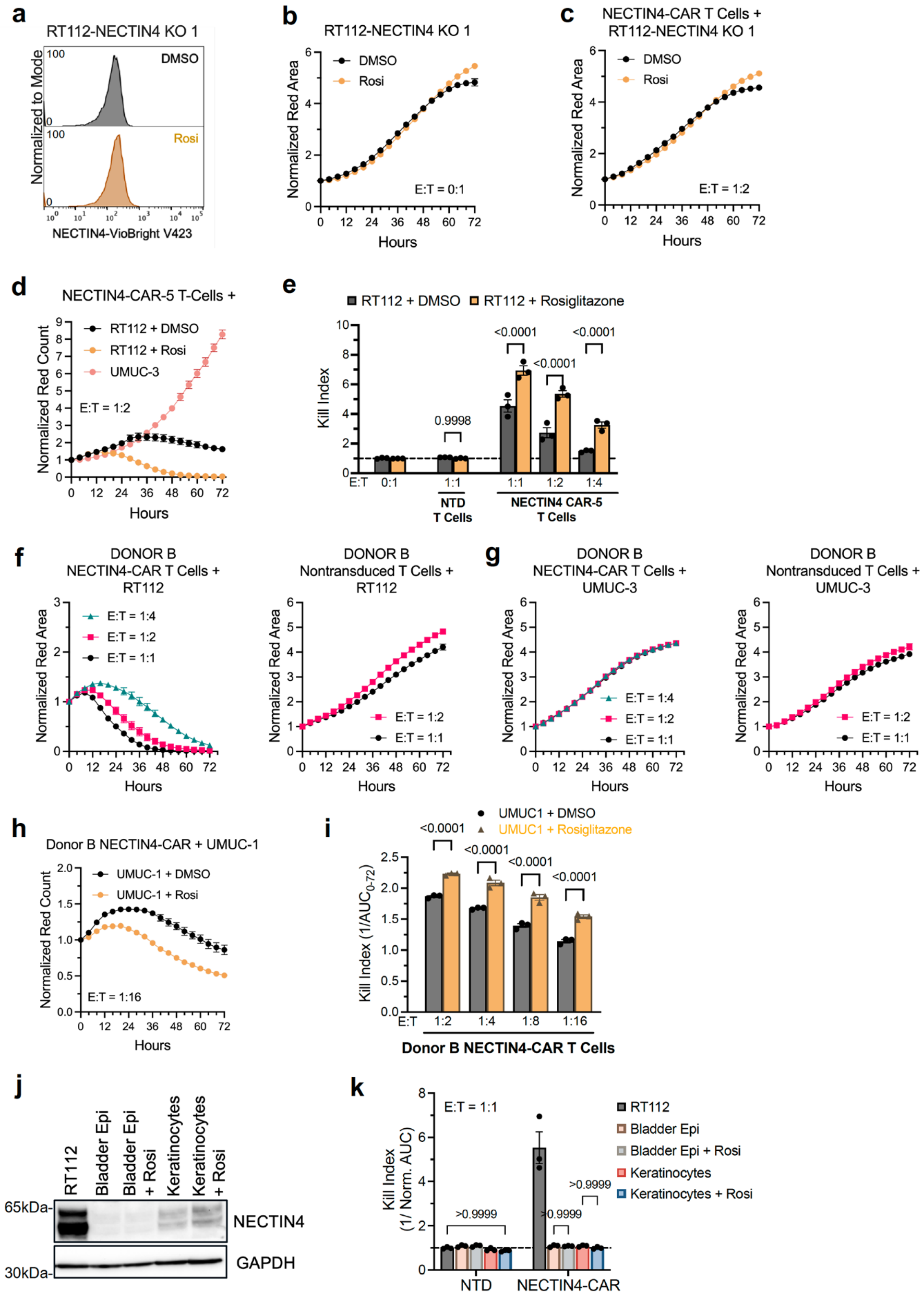

Supplementary Figure 3: Rosiglitazone pretreatment enhances sensitivity to NECTIN4-CAR T cell constructs specifically against UC cells. (**a**) Surface staining for NECTIN4 protein in RT112 NECTIN4 KO cells following treatment with 1 μM rosiglitazone for 72h. Representative growth curves of RT112-NECTIN4 KO cells pretreated for 72h with either DMSO (black) or 1 μM rosiglitazone (gold) in culture alone (**b**) or in co-culture with NECTIN4-CAR T cells (**c**) at an E:T ratio of 1:2. (**d**) Representative growth curves of UMUC-3 cells (red)

and RT112 cells pretreated for 72h with either DMSO (black) or 1 μM rosiglitazone (gold) measured over time upon co-culture with T cells from the primary donor transduced with the NECTIN4-CAR-5 construct shown in Supplementary Fig. 1a. (**e**) Kill index of NECTIN4-CAR-5 T cells against RT112 target cells pretreated for 72h with DMSO control (black) or 1 μM rosiglitazone (gold) across indicated E:T ratios. Data are presented as mean +/- SEM, n=3 biological replicates in each group. (**f**) Growth curves of RT112 target cells co-cultured with (left) NECTIN4-CAR T cells or (right) NTD T cells from a second T cell donor ("Donor B") at indicated E:T cell ratios. (**g**) Growth curves of UMUC-3 target cells co-cultured with (left) NECTIN4-CAR T cells or (right) NTD T cells from a second T cell donor ("Donor B") at indicated E:T cell ratios. (**h**) Representative growth curves of UMUC-1 cells pretreated for 72h with either DMSO (black) or 1 μM rosiglitazone (gold) co-cultured with NECTIN4-CAR T cells from a second T cell donor ("Donor B"). (**i**) Kill index of NECTIN4-CAR T cells from a second T cell donor ("Donor B") against UMUC-1 cells pretreated for 72h with either DMSO (black) or 1 μM rosiglitazone (gold) across indicated E:T ratios. Data are presented as mean +/- SEM, n=3 biological replicates in each group. (**j**) Western blot of NECTIN4 in RT112 cells, normal primary bladder epithelial cells and normal primary keratinocytes treated with or without rosiglitazone. This blot was repeated n=2 independent times with similar results. (**k**) Kill index of NTD and NECTIN4-CAR T cells co-cultured with indicated cell lines shown in (**j**) at E:T ratio of 1:1. Data are presented as mean +/- SEM, n=3 biological replicates in each group. For all growth curves, a representative experiment from n=3 biological replicates is shown and data are presented as mean +/- SEM for each time point. For panels **e, i** and **k**, a two-way ANOVA with Sidak's multiple comparison test was used. Source data are provided as a Source Data file.

Supplementary Figure 4:

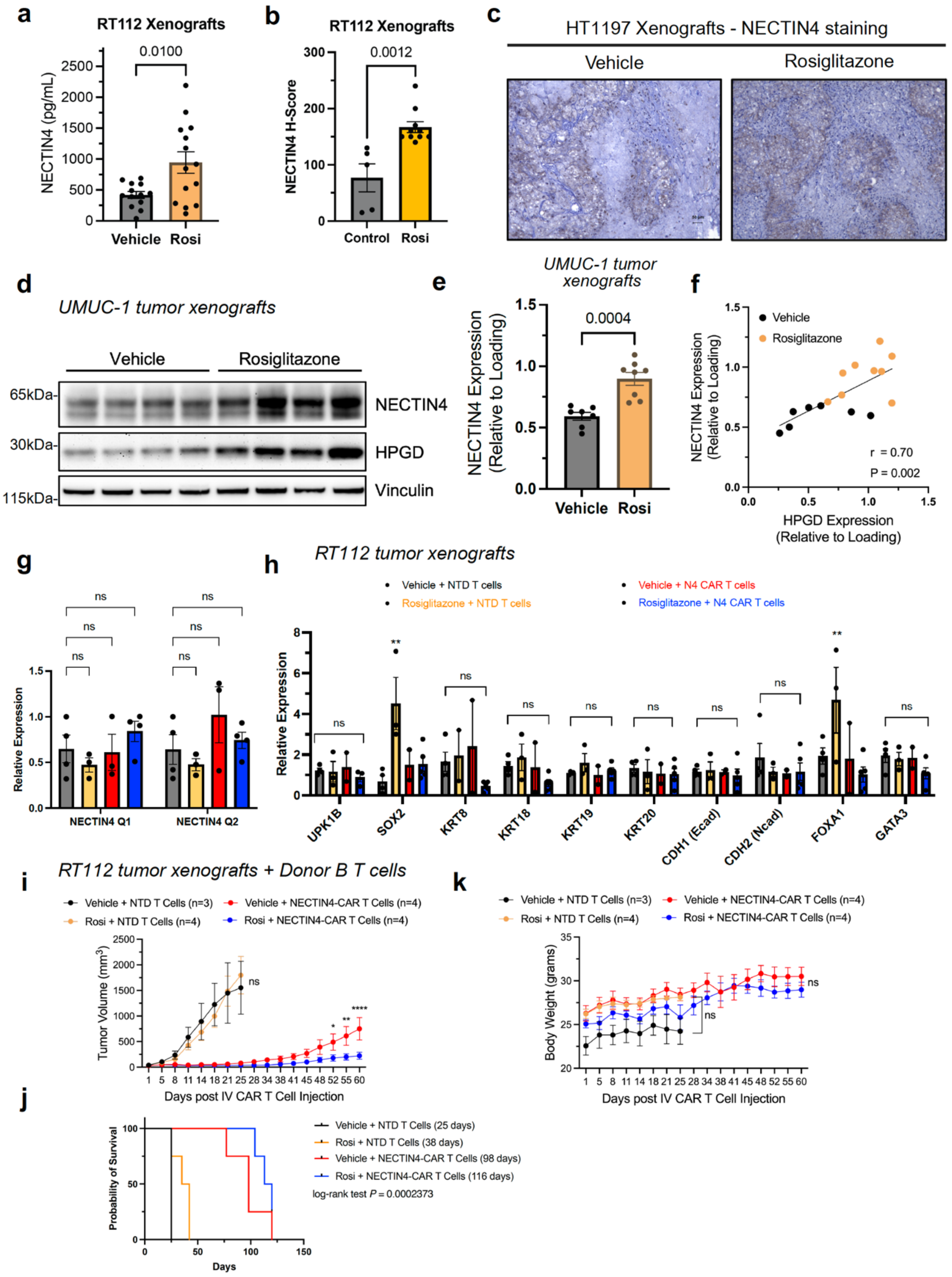

Supplementary Figure 4: Systemic rosiglitazone treatment primes tumor NECTIN4 expression in multiple UC models and enhances anti-tumor efficacy of NECTIN4-CAR T cell therapy *in vivo*. (**a**) NECTIN4 quantification by ELISA from blood of NSG mice bearing RT112 subcutaneous tumor xenografts following treatment with 5 days of vehicle or rosiglitazone. Data are presented as mean +/- SEM, n=13 biological replicates in each group. (**b**) H-scores of membranous NECTIN4 expression on IHC of RT112 tumor xenografts from a single cohort of mice treated with either rosiglitazone or vehicle for 5 days. Data are presented as mean +/- SEM, n=5 biological replicates in control and n=10 biological replicates in Rosi groups. (**c**) Representative images of NECTIN4 staining on HT1197 tumor xenografts from mice treated with vehicle (left) or rosiglitazone (right). Scale

bars = 50 μm. (**d**) Representative western blot for NECTIN4 and HPGD in UMUC-1 tumor xenografts from mice treated with either rosiglitazone or vehicle for 5 days. This blot was repeated n=2 independent times with similar results. (**e**) Quantification of NECTIN4 expression from western blot analysis of UMUC-1 tumors in **(d)**. Data are presented as mean +/- SEM, n=7 biological replicates in Vehicle and n=8 biological replicates in Rosi groups. (**f**) Scatterplot showing NECTIN4 versus HPGD expression from western blot analysis of UMUC-1 tumors in **(d)**. Pearson's correlation is shown for NECTIN4 versus HPGD expression (r = 0.70, *P* = 0.002). Data from n=2 independent cohorts of n=3-5 mice each. **(g)** *NECTIN4* expression as measured by quantitative PCR (qPCR) in RT112 tumors treated with Vehicle + NTD T Cells (gray), Rosi + NTD T cells (yellow), Vehicle + NECTIN4-CAR T cells (red) or Rosi + NECTIN4-CAR T Cells. Data are presented as mean +/- SEM, n=3 biological replicates. **(h)** Expression of indicated luminal and basal/stem-like markers as measured by qPCR in RT112 tumors treated with Vehicle + NTD T Cells (gray), Rosi + NTD T cells (yellow), Vehicle + NECTIN4-CAR T cells (red) or Rosi + NECTIN4-CAR T Cells. Data are presented as mean +/- SEM, n=3 biological replicates in Vehicle+NTD T cells, Rosiglitazone+NTD T cells, n=2 biological replicates in Vehicle+N4 CAR T cells and n=5 for Rosiglitazone+N4 CAR T cells. For panels g and h, ** indicates p=0.01 by two-way ANOVA with Sidak multiple testing correction. ns indicates not significant. (**i-j**) Tumor growth curves **(i)** and Kaplan-Meier survival curve **(j)** of RT112 tumor xenografts after vehicle or rosiglitazone (Rosi) treatment followed by single IV injection of NECTIN4-CAR or NTD T cells from Donor B. For panel **j**, the log-rank test was used. n=3-4 mice in each NTD T cell treated group and n=4 mice in each NECTIN4-CAR T cell treated group. * indicates p=0.0338, ** indicates p=0.001 and **** indicates $p < 0.0001$ at days 52, 55 and 60, respectively, comparing Vehicle+NECTIN4-CAR T (red) vs Rosi+NECTIN4-CAR T (blue). (**k**) Body weights of each group of mice in (**i**) over time. For panels **a**, **b** and **e**, a two-sided, unpaired Student's t test was used. For panels **I** and **k**, a mixed effects model with Sidak's multiple comparison test correction was used. ns indicates not significant**,** NTD indicates non-transduced. For panel **j**, the log-rank test was used to calculate the indicated p value. Source data are provided as a Source Data file.

Supplementary Figure 5:

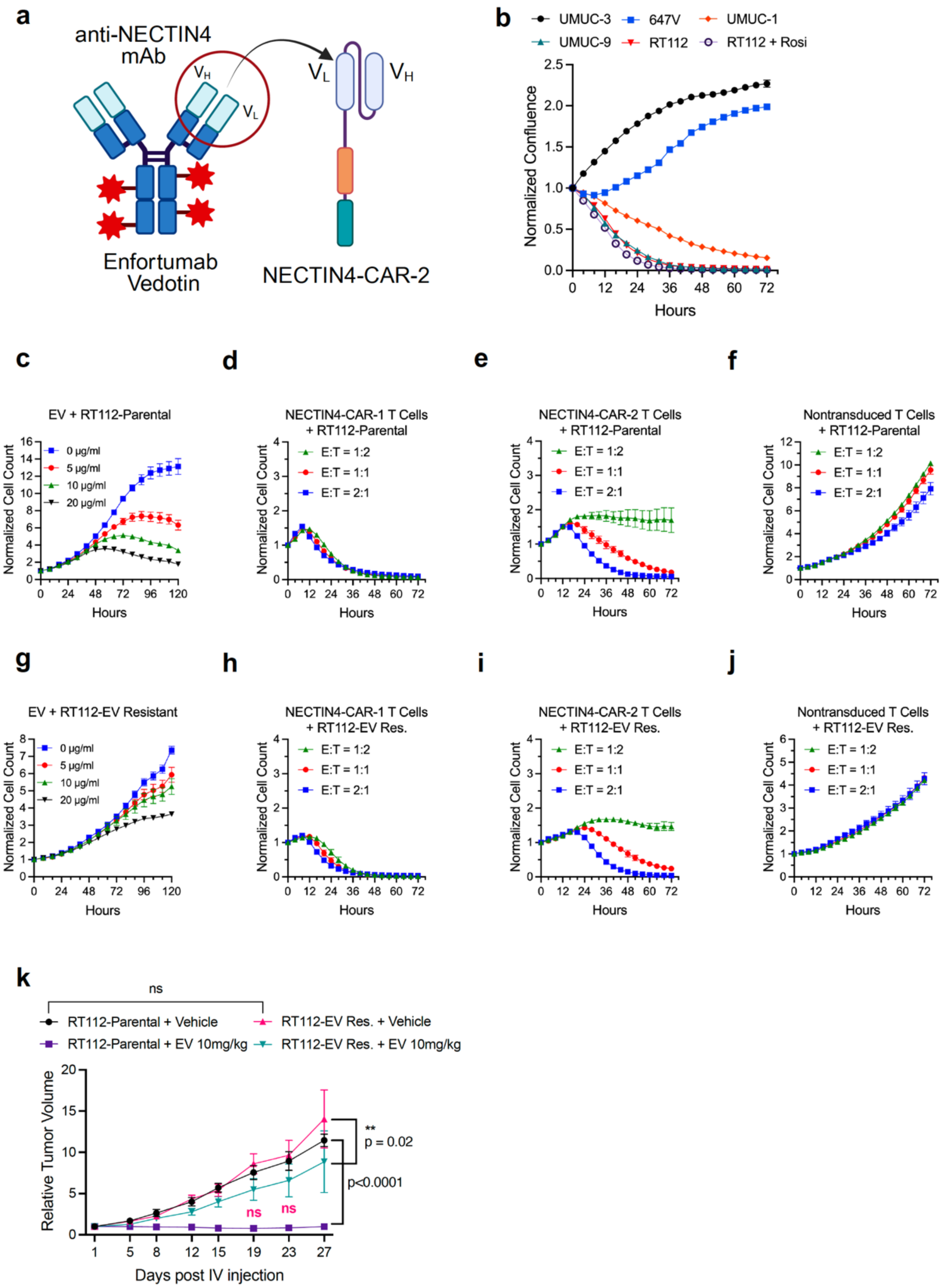

Supplementary Figure 5: NECTIN4 CAR T cells with an enfortumab-derived binder are specific for NECTIN4-expressing UC cells and effective against EV resistant cells. (**a**) Schematic of a second-generation CAR (“NECTIN4-CAR-2”) incorporating the fully humanized NECTIN4-directed scFv derived from enfortumab vedotin (EV), an FDA-approved ADC targeting NECTIN4. Created with BioRender. (**b**) Growth curves of UC cell lines co-cultured with NECTIN4-CAR-2 T cells at an E:T ratio of 1:1. (**c-f**) Representative growth curves of RT112-parental target cells treated with EV (c) or co-cultured with NECTIN4-CAR-1 T cells (d), with enfortumab-derived NECTIN4-CAR-2 T cells (e) or NTD T cells (f) at indicated concentrations or E:T cell ratios. (**g-j**) Representative growth curves of RT112-EV Res. target cells treated with EV (g) or co-cultured with NECTIN4-CAR-1 T cells (h), with enfortumab-derived NECTIN4-CAR-2 T cells (i) or NTD T cells (j) at indicated concentrations or E:T cell ratios. (**k**) Tumor growth curves of RT112-parental and RT112-EVRes. subcutaneous tumor xenografts in NSG mice treated with a single dose of EV (10 mg/kg) or vehicle control. RT112-parental xenografts treated with vehicle (n=8 mice, black), RT112-EV Res. xenografts treated with vehicle (n=4 mice, pink), RT112-parental xenografts treated with EV (n=3 mice, purple), and RT112-EV Res. xenografts treated with EV (n=5 mice, teal). Data are presented as mean +/- SEM, p values are indicated. For panels (**b-j**) a representative experiment of n=3 biological replicates per condition were used and data are presented as mean +/- SEM. For panel **k**, a mixed effects model with Sidak’s multiple comparison test was used. Source data are provided as a Source Data file.

Supplementary Figure 6:

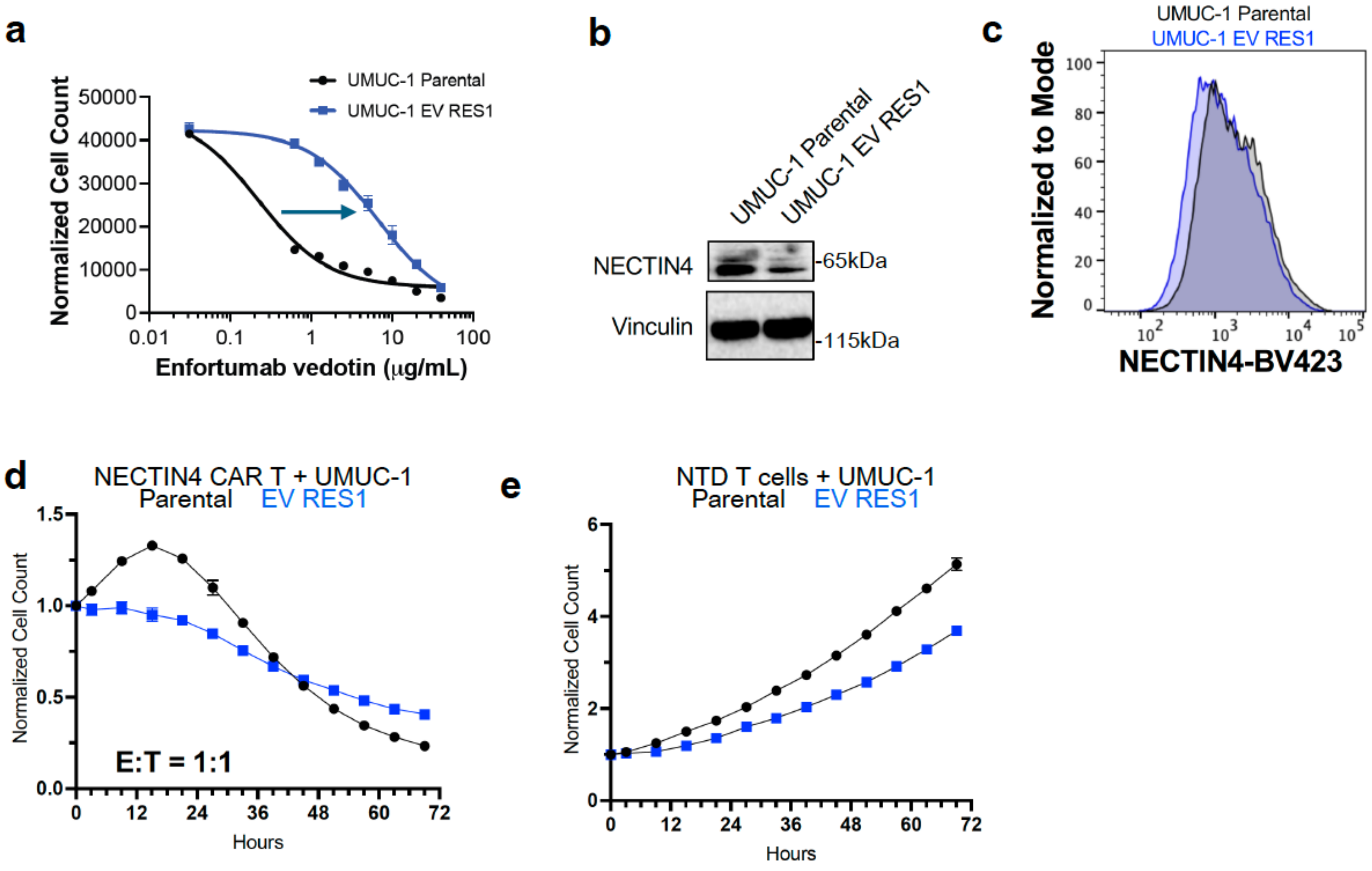

Supplementary Figure 6: NECTIN4 CAR T cell therapy is effective against UMUC-1 EV resistant cells. (**a**) EV dose-response curves for UMUC-1 Parental and EV-resistant (RES1) cells. The IC_{50} in parental and EV RES1 cells is 0.23 µg/ml and 6.8 µg/ml, respectively. (**b**) Western blot showing NECTIN4 levels in the UMUC-1 Parental and EV RES1 whole cell lysates. Vinculin shown as the loading control. This blot was repeated n=2 independent times with similar results. (**c**) Surface NECTIN4 expression in the UMUC-1 Parental and UMUC-1 EV RES1 cells. (**d-e**) Representative curves of UMUC-1 Parental and EV RES1 cells co-cultured with NECTIN4-CAR T cells (**d**) or NTD T cells (**e**) at an E:T of 1:1. For panels **a**, **d**, and **e**, the data are presented as mean +/- SEM, and a representative experiment of n=3 biologically independent experiments performed in technical triplicates is shown. Source data are provided as a Source Data file.

Supplementary Figure 7:

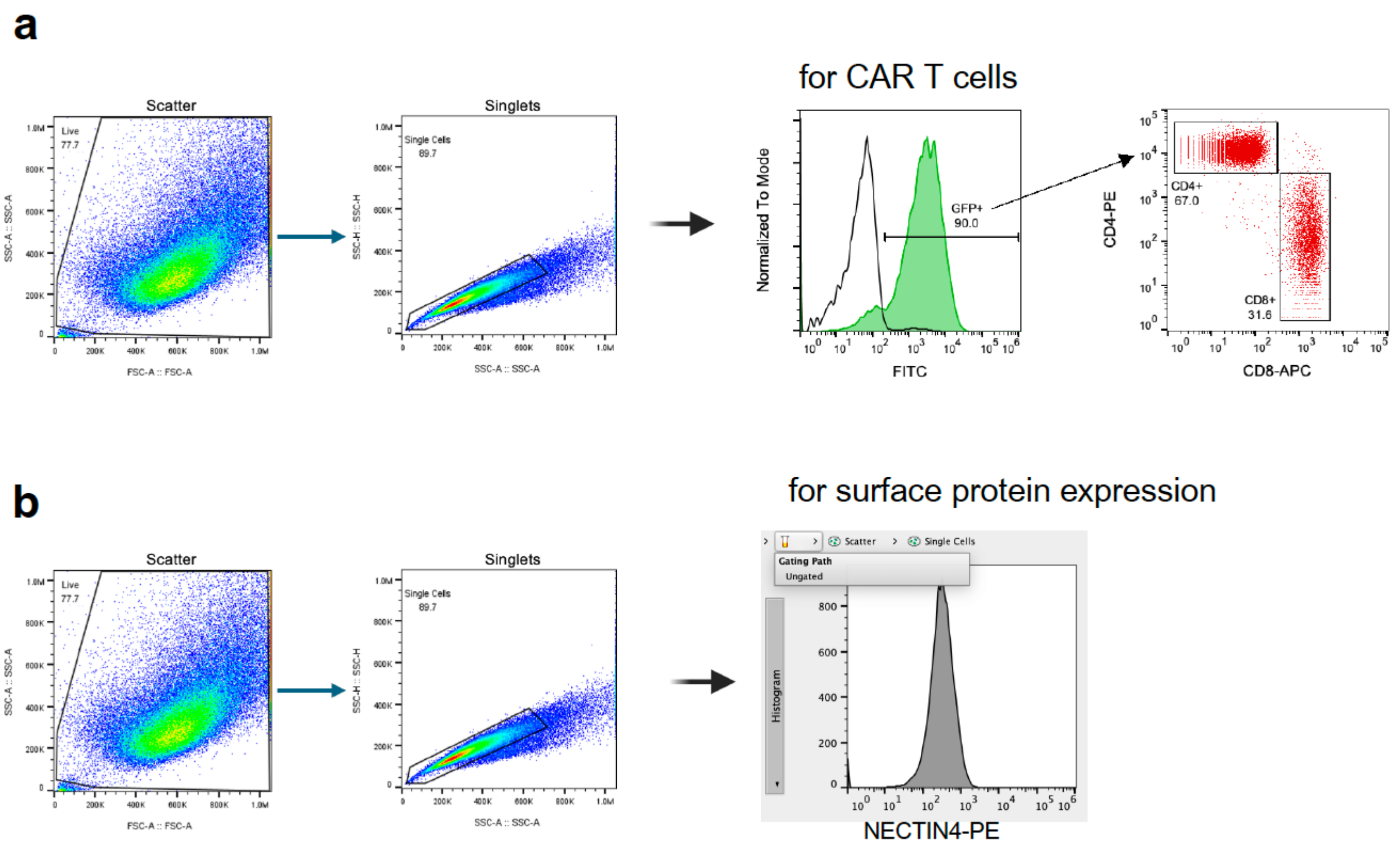

Supplementary Figure 7: Gating strategy: (**a**) Flow cytometry gating strategy for generating and validating NECTIN4 CAR T cells. (**b**) Flow cytometry gating strategy for checking surface protein expression (i.e., NECTIN4 and TROP2) as well as for generating and validating NECTIN4 KO cells**.**

Supplementary Table 1: List of primers used for qPCR.

**Supplementary Table 1**	
**Primer Name**	**Sequence (5' to 3')**
hNECTIN4 qF1	AGGACGCAAAACTGCCCTG
hNECTIN4 qR1	TGAAGCCCGTATTTGGAGTGC
hNECTIN4 qF2	ACGGAGGTCAAAGGCACAAC
hNECTIN4 qR2	GTGGCTGCCCATTCATGCT
hGAPDH qF1	CGACAGTCAGCCGCATCTT
hGAPDH qR1	CCGTTGACTCCGACCTTCA
hGAPDH qF2	ACAACTTTGGTATCGTGGAAGG
hGAPDH qR2	GCCATCACGCCACAGTTTC
hHPRT qF1	GAAAAGGACCCCACGAAGTGT
hHPRT qR1	AGTCAAGGGCATATCCTACAACA
hFOXA1 qF1	GCAATACTCGCCTTACGGCT
hFOXA1 qR1	TACACACCTTGGTAGTACGCC
hGATA3 qF1	GCCCCTCATTAAGCCCAAG
hGATA3 qR1	TTGTGGTGGTCTGACAGTTCG
hKeratin8 qF1	CAGAAGTCCTACAAGGTGTCCA
hKeratin8 qR1	CTCTGGTTGACCGTAACTGCG
hKeratin18 qF1	GGCATCCAGAACGAGAAGGAG
hKeratin18 qR1	ATTGTCCACAGTATTTGCGAAGA
hKeratin19 qF1	TGAGTGACATGCGAAGCCAAT
hKeratin19 qR1	CTCCCGGTTCAATTCTTCAGTC
hKeratin20 qF1	GGACGACACCCAGCGTTTAT
hKeratin20 qR1	CGCTCCCATAGTTCACCGTG
hCDH1 qF1	CGAGAGCTACACGTTCACGG
hCDH1 qR1	GGGTGTCGAGGGAAAAATAGG
hCDH2 qF1	TCAGGCGTCTGTAGAGGCTT
hCDH2 qR1	ATGCACATCCTTCGATAAGACTG
hSOX2 qF1	TGGACAGTTACGCGCACAT
hSOX2 qR1	CGAGTAGGACATGCTGTAGGT
hUPK1B qF1	GACTGCGGAGTGCATCTTCTT
hUPK1B qR1	TGCCCACAAATATGCCGATCC

Supplementary Table 2: List of primers used for ChIP-PCR for predicted PPARG binding sites on the NECTIN4 promoter.

**Supplementary Table 2:**	
**Primer name**	**Primer sequence**
**PPARG Site Primer 1**	Forward TAGCTACGGCTGGGTGTGTA
	Reverse GAGTTCTTGCCTCTCGCACT
**PPARG Site Primer 2**	Forward GTTCCCAGGTTTCTGCCGTC
	Reverse GTTCTACACACCCAGCCGTA
