# Supplementary material for: Modulating the PPARγ pathway upregulates NECTIN4 and enhances chimeric antigen receptor (CAR) T cell therapy in bladder cancer
Source: Nat Commun. 2025 Sep 10;16:8215. doi: 10.1038/s41467-025-62710-0 (PMC12423289; doi:10.1038/s41467-025-62710-0)
Supplement: Supplementary file 2 — Reporting Summary [file 41467_2025_62710_MOESM2_ESM.pdf]

nature portfolio

Corresponding author(s): Jonathan Chou
Last updated by author(s): 6/19/2025

Reporting Summary

Nature Portfolio wishes to improve the reproducibility of the work that we publish. This form provides structure for consistency and transparency in reporting. For further information on Nature Portfolio policies, see our Editorial Policies and the Editorial Policy Checklist.

Statistics

For all statistical analyses, confirm that the following items are present in the figure legend, table legend, main text, or Methods section.

n/a	Confirmed	
☐	☒	The exact sample size (*n*) for each experimental group/condition, given as a discrete number and unit of measurement
☐	☒	A statement on whether measurements were taken from distinct samples or whether the same sample was measured repeatedly
☐	☒	The statistical test(s) used AND whether they are one- or two-sided<br>*Only common tests should be described solely by name; describe more complex techniques in the Methods section.*
☒	☐	A description of all covariates tested
☐	☒	A description of any assumptions or corrections, such as tests of normality and adjustment for multiple comparisons
☐	☒	A full description of the statistical parameters including central tendency (e.g. means) or other basic estimates (e.g. regression coefficient) AND variation (e.g. standard deviation) or associated estimates of uncertainty (e.g. confidence intervals)
☐	☒	For null hypothesis testing, the test statistic (e.g. *F*, *t*, *r*) with confidence intervals, effect sizes, degrees of freedom and *P* value noted<br>*Give P values as exact values whenever suitable.*
☒	☐	For Bayesian analysis, information on the choice of priors and Markov chain Monte Carlo settings
☒	☐	For hierarchical and complex designs, identification of the appropriate level for tests and full reporting of outcomes
☐	☒	Estimates of effect sizes (e.g. Cohen's *d*, Pearson's *r*), indicating how they were calculated

Our web collection on statistics for biologists contains articles on many of the points above.

Software and code

Policy information about availability of computer code

Data collection	Incucyte software, GUI2021 and GUI2023 for cell killing assays; Attune NxT software v4.0 to v4.2 for flow cytometry collection, QuantStudio6 Real-Time PCR Software v1.7.2, Azure300 imager for Western blots
Data analysis	GraphPad Prism, v10.5.0; FlowJo software, v10

For manuscripts utilizing custom algorithms or software that are central to the research but not yet described in published literature, software must be made available to editors and reviewers. We strongly encourage code deposition in a community repository (e.g. GitHub). See the Nature Portfolio guidelines for submitting code & software for further information.

Data

Policy information about availability of data

All manuscripts must include a data availability statement. This statement should provide the following information, where applicable:

- Accession codes, unique identifiers, or web links for publicly available datasets
- A description of any restrictions on data availability
- For clinical datasets or third party data, please ensure that the statement adheres to our policy

No publicly available datasets were used for this study. There are no restrictions on cell line or mouse data availability. Data on NECTIN4 IHC scores from the biopsy samples are available upon request but the biopsy material or FFPE blocks are not available due to patient privacy laws and compliance with HIPAA. All biopsies were collected as part of standard of care, through the MSK IRB# 12-245 and 06-107 protocols for this work.

Research involving human participants, their data, or biological material

Policy information about studies with human participants or human data. See also policy information about sex, gender (identity/presentation), and sexual orientation and race, ethnicity and racism.

Reporting on sex and gender	We do not report on sex or gender in our study. However, we utilized cell lines from women (RT112, HT1376) and men (UMUC3, UMUC1, UMUC9, HT1197). When tumor xenograft experiments were performed, the sex of the cell line was matched to the sex of the mice.
Reporting on race, ethnicity, or other socially relevant groupings	Race, ethnicity or other socially relevant grouping are not included in our study. We did utilize one cell line from an African-American patient (UMUC1).
Population characteristics	Patients with metastatic urothelial carcinoma underwent standard of care biopsies, prior to and after receiving enfortumab vedotin (EV). Patients included men and women.
Recruitment	Patients with metastatic urothelial carcinoma who agreed to standard of care biopsies. There was no specific recruitment initiative.
Ethics oversight	The biopsies were collected as standard of care, through the MSK IRB# 12-245 and 06-107 protocols for this work.

Note that full information on the approval of the study protocol must also be provided in the manuscript.

Field-specific reporting

Please select the one below that is the best fit for your research. If you are not sure, read the appropriate sections before making your selection.

☒ Life sciences ☐ Behavioural & social sciences ☐ Ecological, evolutionary & environmental sciences

For a reference copy of the document with all sections, see nature.com/documents/nr-reporting-summary-flat.pdf

Life sciences study design

All studies must disclose on these points even when the disclosure is negative.

Sample size	No statistical methods were used to predetermine the sample size. We increased the sample size, including the number of cell lines, as much as possible to make conclusions solid and robust.
Data exclusions	Data from our initial rosiglitazone dose-finding experiments in animals were excluded.
Replication	For co-culture killing assays, cells were plated in triplicate per group and biological replicates were performed n=3 times independently, unless otherwise indicated. For rosiglitazone stimulation assays, these were all performed at least n=3 biologically independent times, as indicated. All CAR T assays were performed with 3unique Leukopak/T cell donors. Animal experiments were performed with n=4-5 mice per group, and at least 2 biologically independent experiments, unless otherwise indicated.
Randomization	Plates of cells were randomly assigned to control (DMSO) and Rosiglitazone treatment, and then subjected to co-culture killing assays with indicated conditions randomly. Mice were randomly assigned to received control/Rosiglitazone and control CAR T or N4-CAR T treatments by cage, and treatment groups were assigned by rank ordering tumor sizes whenever possible. Tumor collection at the endpoint was random assigned. For NECTIN4 IHC, each biopsy sample was selected in a random order to assign an H-score.
Blinding	Tumor measurements for in vivo studies were performed in a blinded fashion, as the person taking the measurements did not know which group each mouse belonged to. Cell lines were treated with indicated drugs and read on the Attune cytometry in a blinded manner. ChIP-PCR was performed in a blinded fashion. For NECTIN4 IHC, the pathologist scoring the H-score was blinded to the treatment group and whether the biopsy was taken pre-EV or post-EV treatment. Microscopy images were obtained in a blinded fashion and denoted by pathology accession number.

Reporting for specific materials, systems and methods

We require information from authors about some types of materials, experimental systems and methods used in many studies. Here, indicate whether each material, system or method listed is relevant to your study. If you are not sure if a list item applies to your research, read the appropriate section before selecting a response.

Materials & experimental systems

n/a	Involved in the study
☐	☒ Antibodies
☐	☒ Eukaryotic cell lines
☒	☐ Palaeontology and archaeology
☐	☒ Animals and other organisms
☐	☒ Clinical data
☒	☐ Dual use research of concern
☒	☐ Plants

Methods

n/a	Involved in the study
☒	☐ ChIP-seq
☐	☒ Flow cytometry
☒	☐ MRI-based neuroimaging

Antibodies

Antibodies used

NECTIN4 antibody for flow cytometry, Miltenyi, clone REA967 (lot #1324070142), 1:100; TROP antibody for flow cytometry, Miltenyi, clone REA916, 1:100. GAPDH, Cell Signaling Technology, #2118, clone 14C10, 1:4000; Vinculin, Cell Signaling Technology, #13901, clone E1E9V, 1:2000; NECTIN4, abcam #192033, clone EPR14613-68, 1:1000; PPARγ, Cell Signaling Technology, #2435, clone C26H12, 1:1000; HPGD, R&D Systems, AF5660, 1:1000; FABP4, Cell Signaling Technology, #2120, 1:1000; HRP-anti-rabbit, Cell Signaling Technology, #7074, 1:8000; HRP-anti-goat, abcam, #ab6741, 1:8000; Normal Rabbit IgG, Cell Signaling #3900, 1:100; NECTIN4 for IHC, abcam #192033, clone EPR14613-68, 1:100; Biotinylated anti-rabbit Jackson #111-065-144, 1:1000

Validation

Antibodies were validated per manufacturer's website. For the NECTIN4 antibodies, these were validated by creating a NECTIN4 KO cell line and validating that the signal was abolished in the NECTIN4 KO cell line.

Eukaryotic cell lines

Policy information about cell lines and Sex and Gender in Research

Cell line source(s)

Cells were obtained from the UCSF Cell Culture Facility, who originally obtained them from the ATCC (UMUC3, 647V, HT1197, HT1376). Cells were also obtained from Dr. Peter Black (RT112, UMUC1, UMUC9, from the Univ. of British Columbia) who previously obtained them from The UBC40 cell line resource and Dr. Peter McConkey. Normal bladder epithelial A/T/N cells (PCS-420-010) and normal skin keratinocytes (PCS-200-011) were purchased from ATCC; additional source of keratinocytes were a gift from Dr. Bahram Razani. T cells were isolated from a LeukoPak, which was purchased from STEMCELL Technologies (obtained through their IRB protocol from human donors). Two different T cell donors were used for the study.

Authentication

Cells were authenticated by STR profiling.

Mycoplasma contamination

Cells were negative for mycoplasma and routinely tested every 3 months.

Commonly misidentified lines
(See ICLAC register)

None.

Animals and other research organisms

Policy information about studies involving animals; ARRIVE guidelines recommended for reporting animal research, and Sex and Gender in Research

Laboratory animals

NOD-SCID-Gamma (NSG) male and female mice, 8-14 weeks old were used for in vivo studies

Wild animals

N/a

Reporting on sex

N/a

Field-collected samples

N/a

Ethics oversight

Animal studies were approved on IACUC protocol AN202104 and AN198293.

Note that full information on the approval of the study protocol must also be provided in the manuscript.

Clinical data

Policy information about clinical studies
All manuscripts should comply with the ICMJE guidelines for publication of clinical research and a completed CONSORT checklist must be included with all submissions.

Clinical trial registration

N/A

Study protocol	MSK IRB# 12-245 and 06-107 protocols for research tissue analysis of biopsies performed as standard of care.
Data collection	The biopsies were collected as standard of care, through the MSK IRB# 12-245 and 06-107 protocols for this work, between 2019-2021.
Outcomes	N/A

Plants

Seed stocks	N/A
Novel plant genotypes	N/A
Authentication	N/A

Flow Cytometry

Plots

Confirm that:

☒ The axis labels state the marker and fluorochrome used (e.g. CD4-FITC).

☒ The axis scales are clearly visible. Include numbers along axes only for bottom left plot of group (a 'group' is an analysis of identical markers).

☒ All plots are contour plots with outliers or pseudocolor plots.

☒ A numerical value for number of cells or percentage (with statistics) is provided.

Methodology

Sample preparation	Cells were washed with D-PBS and then 1ml of TrypLe or Trypsin (0.25%) was added for 5 min at 37C. Cells were collected with complete media (+10% FBS) and washed with D-PBS x 2. Cells were then stained for indicated targeted with the indicated primary antibody on ice for 30-60 minutes, and then washed again in D-PBS x2 and resuspended in FACS buffer (PBS + 2% FBS) and kept on ice until they were ready to be analyzed or sorted. If cells were clumpy, they were first passed through a 40 or 70 micron filter prior to starting the run. All cells that were ran through the sorter were passed through a 40micro filter.
Instrument	Attune NxT (for analysis) and BD Fusion (for sorting)
Software	Attune NxT v4.0 to v4.2. BDFACS Diva software v9.0
Cell population abundance	For cells labeled with NucLightRed, cells were sorted at least 2 times to achieve a homogenously and uniformly red fluorescent population. For analysis, at least 30,000 events were collected in the singlet gate.
Gating strategy	Scatter gate (SSC vs FSC) and then single gate (SSC-H vs SSC-A) and then the fluorescent population.

☒ Tick this box to confirm that a figure exemplifying the gating strategy is provided in the Supplementary Information.
