# Supplementary material for: Modulating the PPARγ pathway upregulates NECTIN4 and enhances chimeric antigen receptor (CAR) T cell therapy in bladder cancer
Source: Nat Commun. 2025 Sep 10;16:8215. doi: 10.1038/s41467-025-62710-0 (PMC12423289; doi:10.1038/s41467-025-62710-0)

Fig. 1b

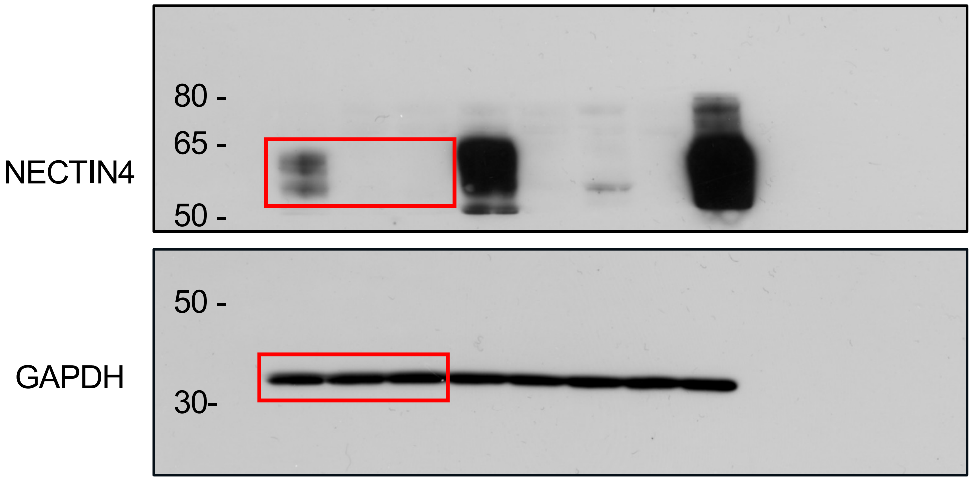

Fig. 1h

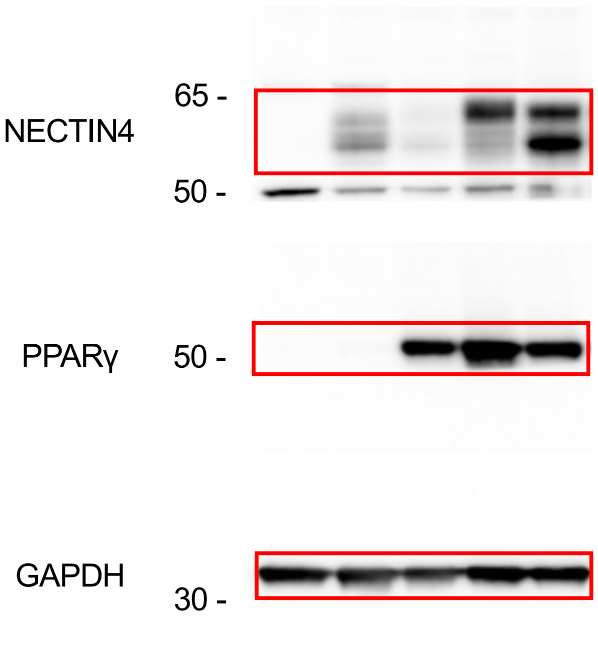

Fig. 2b

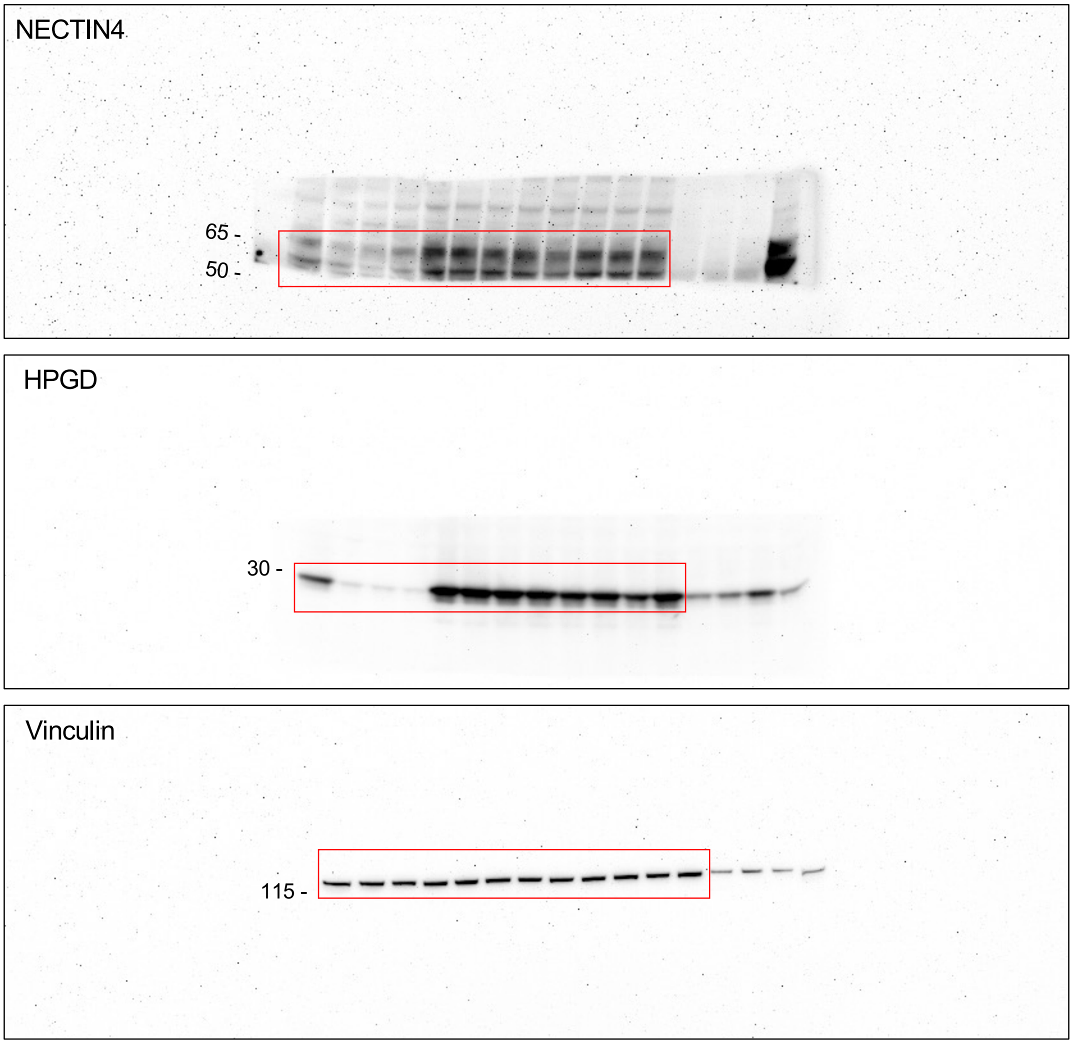

Fig. 2e

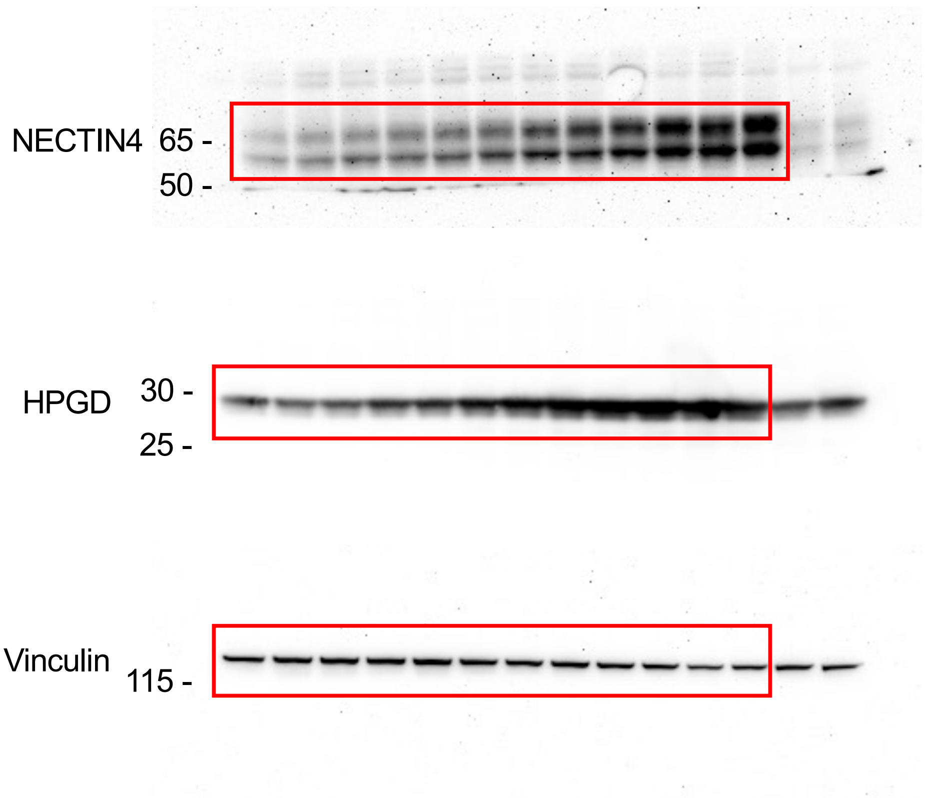
NECTIN4 65 -
50 -
HPGD 30 -
25 -
Vinculin 115 -

Fig. 2i

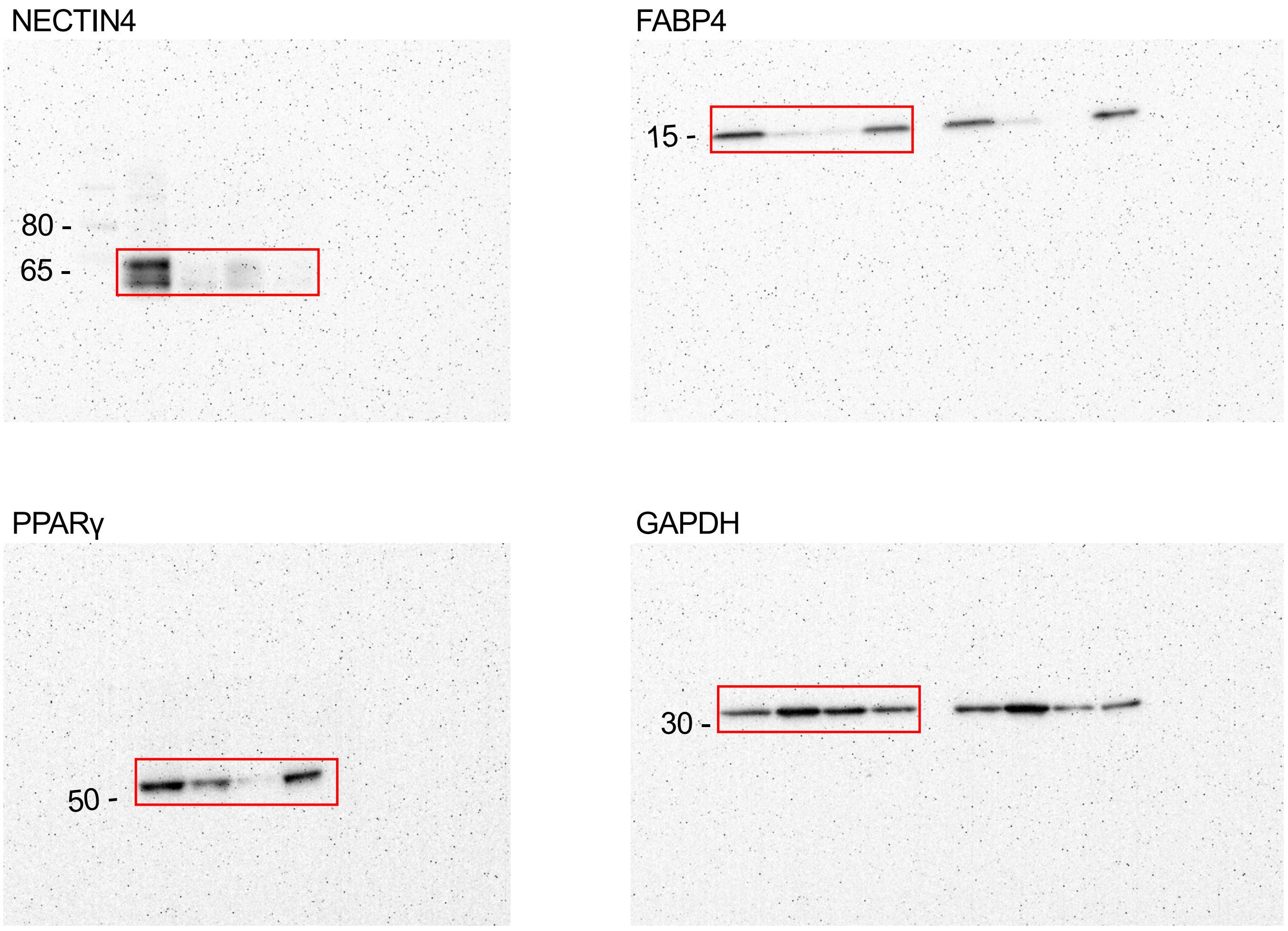
NECTIN4
80 -
65 -
FABP4
15 -
PPARγ
50 -
GAPDH
30 -

Fig. 2k

Primer 1

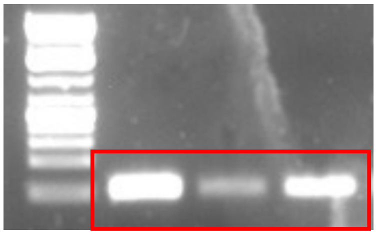

Primer 2

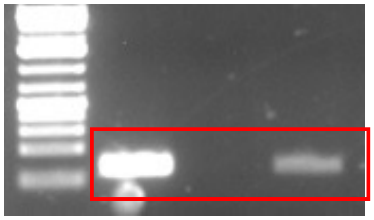

Fig. 4b

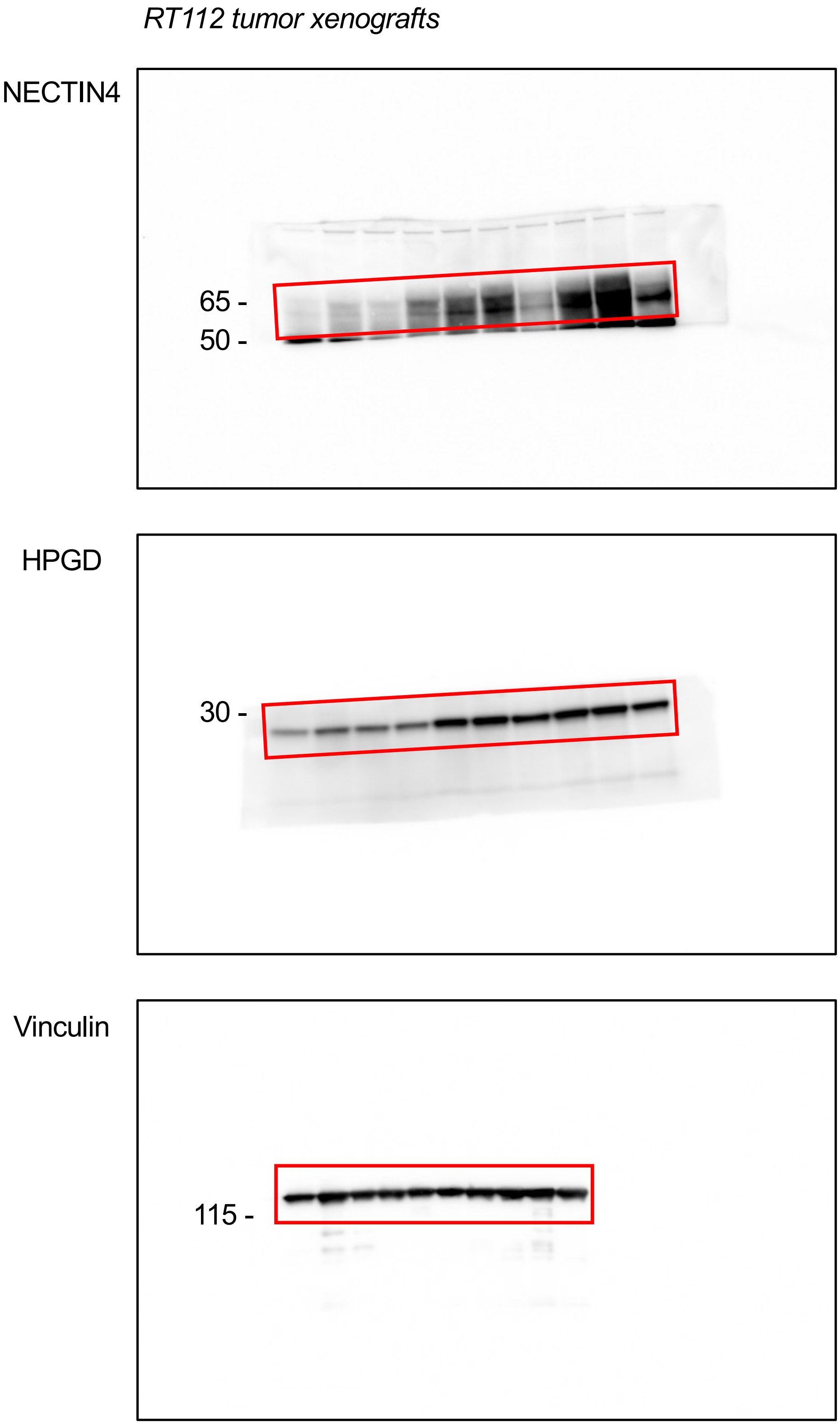

Fig. 4h

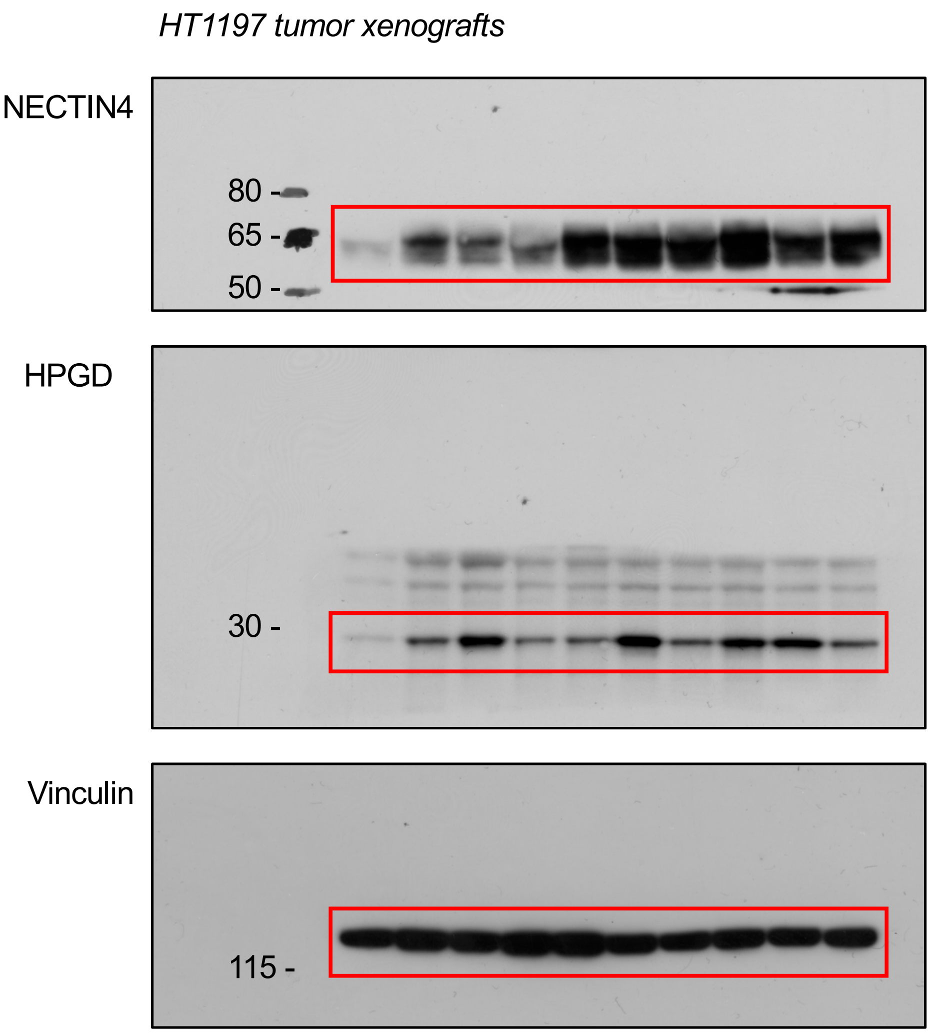

HT1197 tumor xenografts
NECTIN4
80 -
65 -
50 -
HPGD
30 -
Vinculin
115 -

Fig. 5c

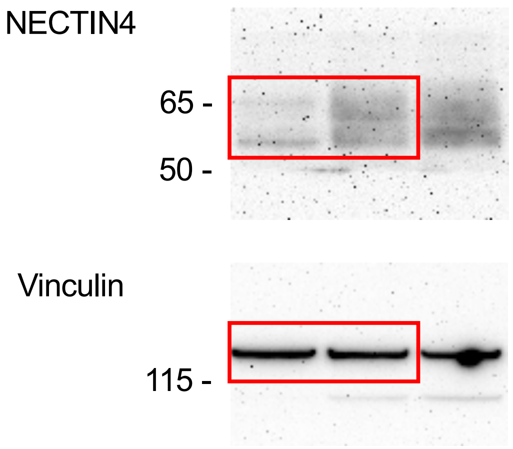

Sup. Fig. 1d

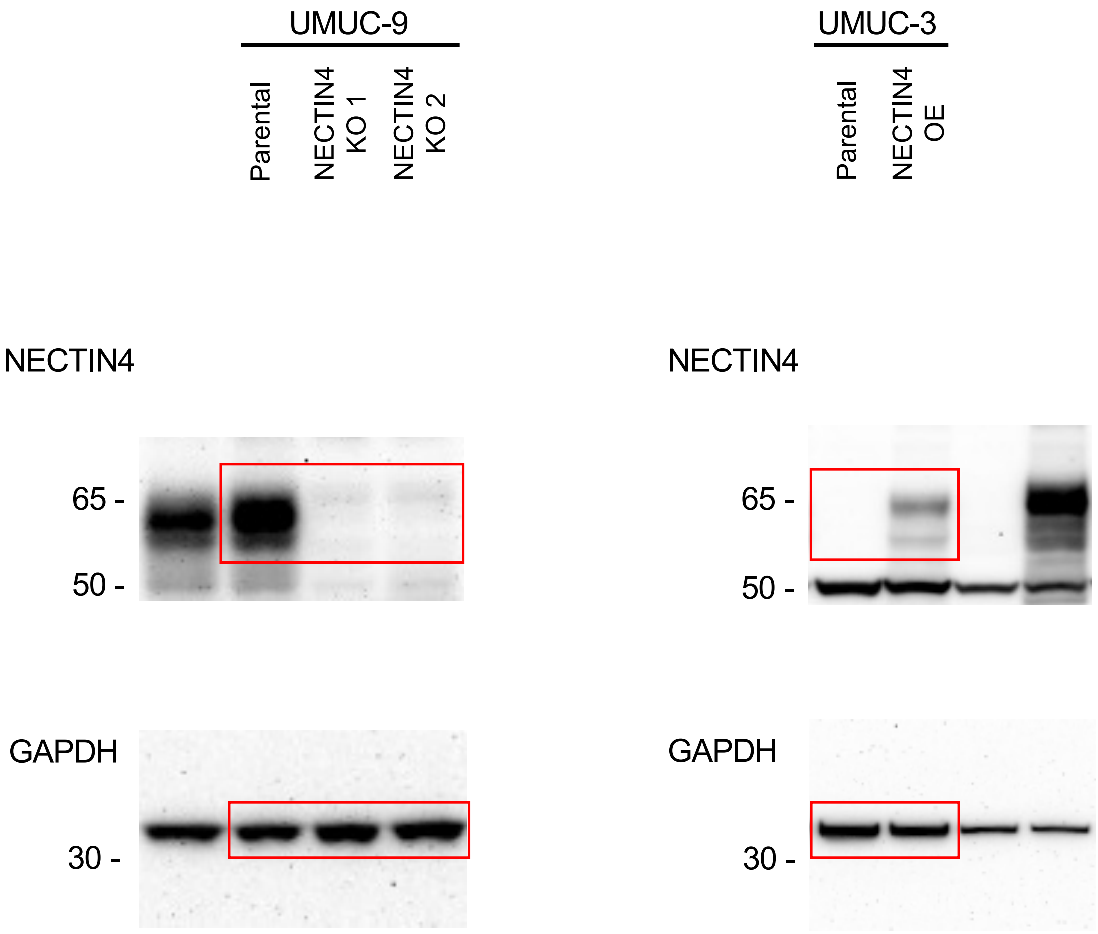

Sup. Fig. 1m

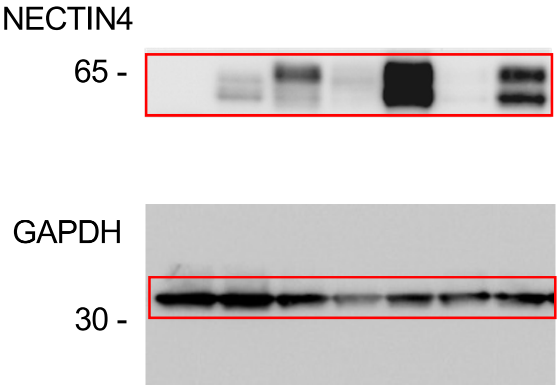

Sup. Fig. 2b

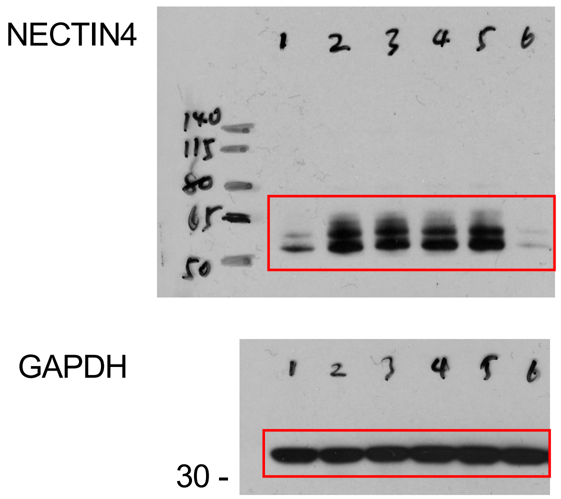

Sup. Fig. 2c

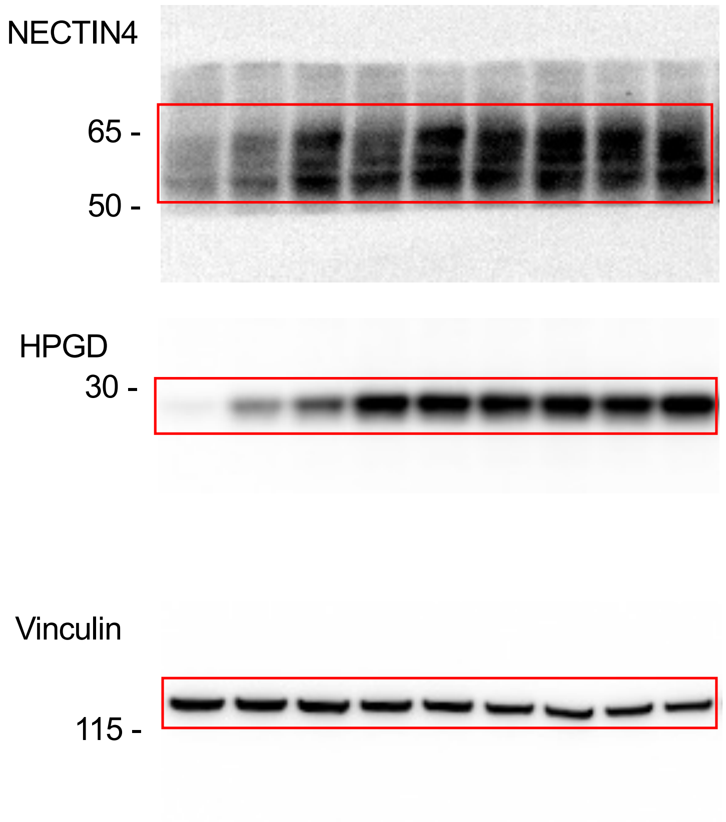

Sup. Fig. 2e

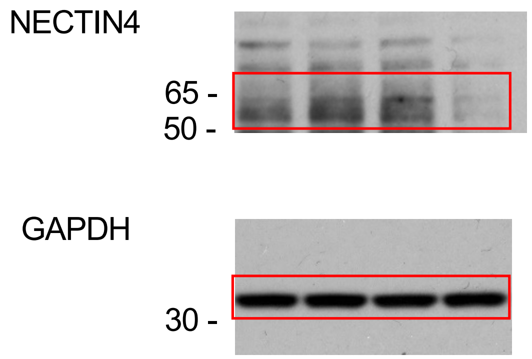

Sup. Fig. 2g

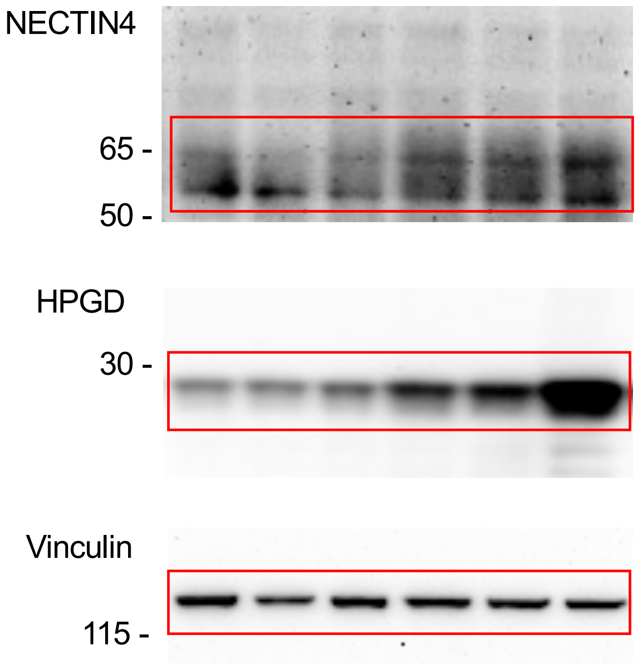

Sup. Fig. 2j

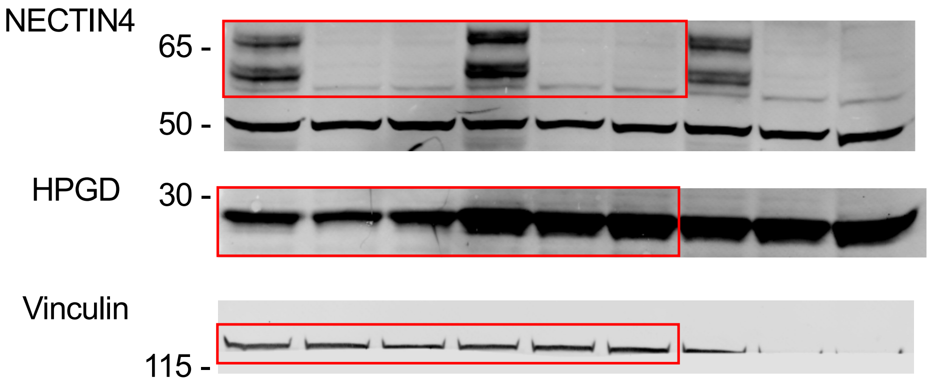

Sup. Fig. 3j

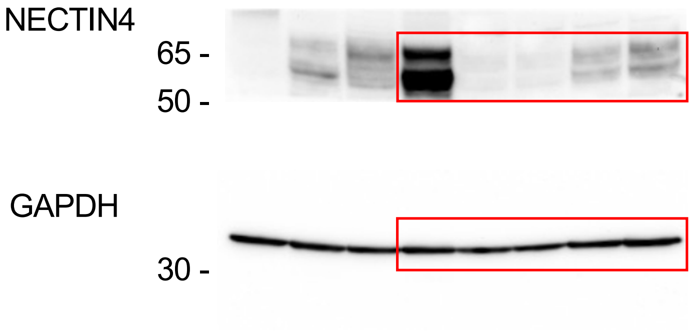

Sup. Fig. 4d

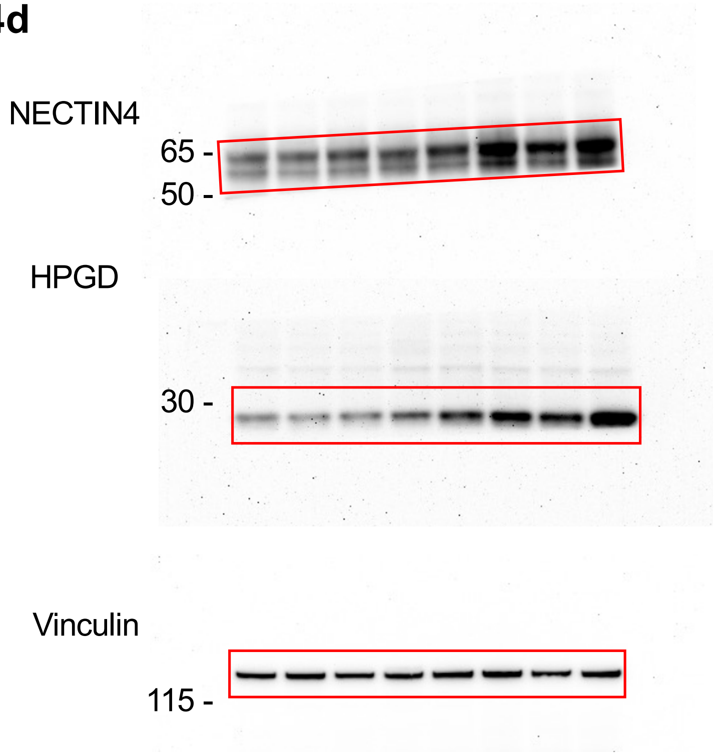

Sup. Fig. 6b

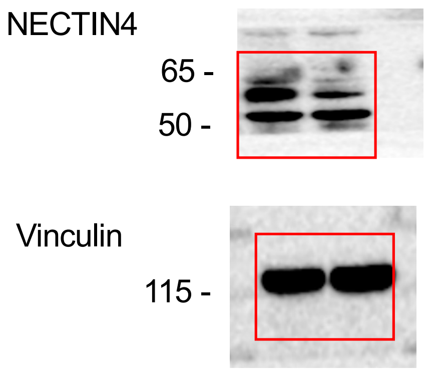

Supplement: Supplementary file 5 — Source Data Blots [file 41467_2025_62710_MOESM5_ESM.pdf]
